# Optimizing Systematic Endoscopic Lymph Node Staging in Non-Small Cell Lung Cancer: Proposals for Integrating the 9th Edition N-Staging Classification into Clinical Practice

**DOI:** 10.3390/cancers18142208

**Published:** 2026-07-09

**Authors:** Daniel P. Steinfort, Matthew Evison

**Affiliations:** 1 Department of Respiratory and Sleep Medicine, Royal Melbourne Hospital, 300 Grattan Street, Melbourne, VIC 3050, Australia; 2 Faculty of Medicine, Dentistry & Health Sciences, University of Melbourne, Parkville, VIC 3010, Australia; 3 Lung Cancer & Thoracic Surgery Directorate, Wythenshawe Hospital, Manchester University NHS Foundation Trust, Manchester M23 9LT, UK; m.evison@nhs.net; 4 Manchester Academic Health Science Centre, Faculty of Biology, Medicine & Health, University of Manchester, Manchester M13 9PL, UK

**Keywords:** bronchoscopy, lung cancer, staging, synoptic reporting, quality, chemoradiation

## Abstract

Systematic endoscopic mediastinal lymph node staging is imperative to most accurately define the extent of LN involvement in patients with locally advanced (cN2) NSCLC. It provides the most accurate nodal information in comparison to imaging techniques and is the most likely investigation to differentiate N2a from N2b disease. Accumulation of evidence to validate the prognostic, and potentially the predictive value of accurately defining N2a and N2b N-status will be supported by more consistent and more comprehensive mediastinal endoscopic staging in this group, aided by more detailed (synoptic) reporting. We identify proposed solutions for enhancement of systematic staging in patients with N2 NSCLC, including routine performance of systematic lymph node assessment, with sampling of all LN > 6 mm, addition of EUS-B-FNA where possible, and use of synoptic reporting to reduce variance in care and enable performance monitoring.

## Simple Summary

Checking the lymph nodes in the center of the chest (the mediastinum) is a standard and essential step for patients with early-stage lung cancer. Emerging evidence shows it is just as vital for patients with more advanced lung cancer because it helps doctors map out the best possible treatment plan. Accurate checking helps doctors precisely target radiation therapy, spot cancer that has spread too far for surgery (steering patients toward safer non-surgical treatments), and make sure patients who *can* be cured by surgery aren’t accidentally denied it. Using a camera scope (endoscopy) to check these nodes gives much more accurate information than regular scans. It is the best way to tell if the cancer is in just one area (N2a) or multiple areas (N2b), which helps doctors personalize patient care. The latest international cancer guidelines (the 9th edition) emphasize this precise checking to improve the quality and consistency of care. To make sure these checks are done perfectly every time and to minimize variation between hospitals, the authors propose several solutions including using standardized, checklist-style medical reports (synoptic reporting) and regularly tracking doctors’ performance.

## 1. History of the N-Staging Classification in Lung Cancer

The critical influence of lymph node (LN) involvement on lung cancer prognosis has been established for over 65 years [1] and was recognized with the inclusion of three “N” category descriptors (N0—no nodal metastases, N1—nodal metastases at the ipsilateral hilum, and N2—nodal metastases in the mediastinum) in the original Union for International Cancer Control (UICC) Tumour Node Metastasis (TNM) classification for lung cancer published in 1966 [2].

Through early revisions to the TNM staging classification for Non-small Cell Lung Cancer (NSCLC), N2 remained a single category for describing mediastinal involvement. In the 4th edition of the TNM classification for lung cancer, it was recognized that a further subdivision of the N2 category into N2 and N3 (with N3 representing nodal metastases in the contralateral mediastinum/hilum), ref. [3], provided greater accuracy in defining prognosis and was a first step into sub-dividing the extent of mediastinal LN involvement for better prediction of outcome.

During the development of the 7th edition TNM staging edition, it was recognized that there were international differences in the terminology and “maps” used to describe intra-thoracic lymph nodes within lymph node staging. To achieve global uniformity the International Association for the Study of Lung Cancer (IASLC) proposed a new lymph node map which reconciled differences between the pre-existing maps and provided precise anatomic definitions for all lymph node stations [4]. Following this unification, the next requirement to progress our description of lymph node involvement was to examine the difference between single-station and multi-station N2 NSCLC. There were long-held beliefs and published evidence suggesting these were two distinct prognostic groups [5]. Prospective data on single-station versus multi-station N2 were collected within the IASLC staging databases as part of the development of the 9th edition TNM and resulted in the accumulation of robust clinical evidence validating the use of this subdivision to more accurately predict prognosis.

As a result, the 9th Edition TNM Classification [6] formally implemented these descriptors (single-station N2 = N2a, multi-station N2 = N2b), upgrading them from optional data points to core staging components. These data demonstrated a clear, stepwise deterioration in 5-year overall survival moving from N2a (single-station) to N2b (multi-station), providing the statistical justification to recognize the differing prognosis inferred by this additional subclassification of N2 lung cancer. Thus, the transition to the 9th Edition of the TNM Classification for Lung Cancer emphasizes an important development in nodal staging that can support more informed discussions with our patients, decision-making and research into optimal management strategies. This enhanced prognostic information has arrived at an optimal time with substantial advances in the curative-intent multimodality treatment landscape for stage II/III NSCLC.

## 2. Integrating the 9th Edition N-Staging Classification into Clinical Practice

The UICC describes the TNM cancer staging classification as “the common language in which oncology health professionals can communicate on the cancer extent for individual patients as a basis for decision making on treatment management and individual prognosis” [7]. Thus, TNM staging provides global standardization as well as accurate prognostication to support high-quality discussion with patients and to support treatment considerations. However, the TNM stage does not act as the sole driver of treatment decisions. This is an important distinction in the context of N2a and N2b NSCLC.

It is critical to note that while clearly providing ***prognostic*** information, no published evidence establishes delineation of N2a from N2b as having ***predictive*** value in identifying a preferred treatment based on N-status. Multiple randomized controlled trials over several decades that compared surgical multimodality treatment versus non-surgical multimodality treatment in stage III-N2 NSCLC failed to identify an advantage in overall survival of one treatment regimen over another [8]. We note that recruitment into surgical versus non-surgical multimodality RCTs is extremely challenging and a recent feasibility trial reported substantial concerns about the feasibility to run a large-scale RCT in this area [9], questioning whether the optimal treatment in this cohort of patients will ever be truly answered within a research study.

Despite this, a narrative has developed, even recognized within certain lung cancer guidelines, that N2a represents a surgical entity whereas N2b represents disease that should be managed with non-surgical modalities [10]. In fact, published evidence confirms that selected cases of N2a can very reasonably be treated with non-surgical multimodality treatment and selected cases of N2b can be managed with surgical multimodality treatment. This is particularly important as the TNM classification is based on the number of N2 lymph node stations involved (single versus multiple) but does not take into account lymph node size (bulky versus non-bulky) or the relationship to adjacent structures including other lymph nodes (invasive or conglomerate lymph nodes versus non-invasive). Therefore, a T2a single-station N2 node could be bulky and invasive and multi-station N2b lymph nodes could be non-bulky and non-invasive. The treatment decisions in these scenarios would likely be based on the size and relationship to adjacent structures rather than the N2a versus N2b classification.

There are numerous publications that question the use of single-station N2 versus multi-station N2 as a treatment-defining factor, particularly using this as a distinction for surgical management versus non-surgical. The PACIFIC trial [11] was a trial of “unresectable” stage III NSCLC yet 50% of patients had stage IIIA disease—a stage group that is potentially highly likely to be technically resectable and include single-station N2 disease. Nevertheless, the survival benefit following consolidation IO following definitive chemoradiation was clear. Furthermore, neoadjuvant and peri-operative chemo-immunotherapy have become a standard of care for non-driver mutated stage II/IIINSCLC. Recently, Diaz et al. undertook a meta-analysis of the sub-group of patients from the AEGEAN (10% patients had multi-station N2), Checkmate 77T (15% of patients had multi-station N2), KEYNOTE 671, and NADIM II trials (39% of patients had multi-station N2 disease). The pooled hazard ratio for event-free survival (EFS) for N2-single station tumours was 0.58, virtually identical to N2-multi station disease at 0.59 [12]. This suggested the substantial benefit inferred by neoadjuvant and peri-operative chemo-immunotherapy over chemotherapy alone in surgical multimodality; curative intent treatment for NSCLC may be similar, regardless of single- versus multi-station N2 status, questioning the use of this as an eligibility for surgical treatment.

In summary, while single-station versus multi-station N2 disease is not itself the determinant of treatment, it is an important and powerful predictor of prognosis. Differentiation of N2a from N2b involvement provides the optimal, standardized description of the extent of mediastinal lymph node metastases to inform patient discussions and support treatment discussions and also provides an opportunity to research optimal treatment in N2a and N2b. The accurate differentiation of single-station from multi-station N2 involvement in NSCLC requires consistent performance of systematic endoscopic mediastinal staging. However, limited evidence on practice in minimally invasive NSCLC staging exists, but consistent findings among studies describing staging practice recognize a wide variation in practice with a minority of centres consistently performing systematic staging [12,13,14]. Furthermore, while quality indicators for EBUS have been proposed, including that frequency of systematic staging in patients staged cN1/2/3 exceed 95% [15], there are currently no metrics to assess the quality of systematic mediastinal staging, and this will be explored further below.

## 3. Limitations of Endoscopic Systematic Staging in N2 NSCLC

A major limitation of systematic endoscopic staging in differentiating N2a from N2b is the inherent imperfect accuracy of EBUS-TBNA. Sampling of pathologic mediastinal LN is associated with excellent diagnostic sensitivity; however, meta-analyses demonstrate reduced accuracy in assessment of radiologically normal LN, with a consistent, small proportion of lymph nodes that are normal on CT, PET and EBUS in patients with lung cancer found to contain occult metastases [16]. Significant rates of upstaging following systematic endoscopic LN staging are noted for all of cN0/1/2a/2b [17]. Highlighting the quality gap in differentiating N2a from N2b disease, recent studies note post-operative pathologic upstaging to pN2b in one third of 115 patients staged cN2a following pre-operative systematic EBUS staging who then underwent up-front surgical resection [18]. This study by Craig and colleagues eloquently identified the limitations of EBUS staging in describing the reasons for missed N2b findings, including:

i. LN stations inaccessible to EBUS;
ii. False-negative TBNA;
iii. Accessible lymph nodes not sampled during systematic staging EBUS.

It is noteworthy that of these three groups, the failure to sample assessable LN (iii) was the commonest (40% of missed N2b cases), and the authors consider this the easiest to immediately address through improvement in procedural practice (see “proposed solutions” below).

The potential impact of comprehensive sampling in patients with locally advanced NSCLC disease during endoscopic mediastinal staging is well-illustrated by findings from the SEISMIC study. The SEISMIC study was a prospective multi-centre international clinical trial examining the impact of systematic mediastinal LN staging to guide radiation planning in patients with locally advanced NSCLC considered for radical dose radiotherapy (in combination with chemotherapy) [19]. It demonstrated a high rate of detection of PET-occult disease (13%) in cN1/2/3 patients, and crucially demonstrated the clinical implications of differentiating N2a from N2b (and N3) disease—in silico dosimetry studies showed the median volume of EBUS-detected PET-occult lymph node metastases receiving the prescription dose of 60 Gy was only 10·1%, indicating a near-universal probability of treatment failure if radiation fields were planned solely on the basis of PET findings (as is currently recommended by international guidelines [20]).

More detailed information illustrating the importance of systematic staging for differentiation of cN2a from cN2b NSCLC is presented in a post hoc analysis of the SEISMIC study published in this edition of *Cancers* [21]. Analysis of sampling patterns confirms detection of PET-occult disease in 18 of 92 (20%) patients *where sampling of at least one radiologically normal LN was performed*. Among 76 participants staged cN2a by PET, EBUS identified PET-occult metastases in seven (9%), with four (5%) upstaged to cN2b, and three (4%) to cN3, emphasizing the clinical importance of systematic staging for differentiation of N2a from N2b (and N3) disease—additional information does not need to result in altered treatment modality to have a major impact on treatment planning and clinical outcomes.

Systematic staging to differentiate N2a from more advanced NSCLC disease (N2b/N3) in the setting of curative intent radiation may be considered analogous to long-standing efforts to promote comprehensive intra-operative lymph node staging in patients undergoing resection of NSCLC [22].

The impact of sub-optimal LN staging in this cohort is clearly demonstrated in the IASLC Lung Cancer Staging Project Residual Tumour Descriptors for Non-Small Cell Lung Cancer [23]. Uncertain residual tumour status [R(un)] was recognized where the highest mediastinal LN resected was positive for metastatic involvement, with this group demonstrating median survival 50 months, compared to 70 months for patients with benign findings at the highest LN station resected [R0] [24].

While residual tumour status (and pN status) is routinely established following resection via intra-operative lymph node sampling/dissection, patients receiving chemoradiation or neoadjuvant chemo-immunotherapy up front cannot have confirmatory pathologic staging completed. In this group, the most thorough determination of N-stage will be via endoscopic lymph node staging. Routine sampling of pathologic LN will inevitably fail to explore the possibility of PET-occult disease, analogous to the R(un) patient group described by the IASLC (above). Where LN ≥ 6 mm remains unsampled endoscopically, these patients similarly have an uncertain status of mediastinal involvement—endoscopic R(un). Similarly, patients undergoing systematic mediastinal staging, with sampling of all LNs at higher stations than observed on PET, may be considered endoscopic R0 (eR0). The concept of eR0 is interesting and novel and worthy of further investigation as a potential quality indicator for staging EBUS.

International guidelines have recommended systematic endoscopic mediastinal staging in early-stage NSCLC since 2015 for patients with any clinicoradiologic risk factors associated with elevated rates of post-operative nodal upstaging, including tumour size > 3 cm, central position of primary tumour, and cN1 on non-invasive imaging [25]. More recent evidence and consensus statements strongly support extending this practice to patients staged cN2, and the move to the 9th Ed TNM provides yet more impetus to refine staging practice in this patient group. Recognizing, as discussed above, that there are limitations to the accuracy of endoscopic staging for differentiation of N2a from N2b disease, we have proposed six solutions to improve minimally invasive staging of T(any) N2 M0 NSCLC.

## 4. Proposed Solutions to Enhance Accuracy of Endoscopic Staging

### 4.1. For All EBUS Centres

#### 4.1.1. Routine Performance of Systematic Mediastinal Staging in Patients with cN1-3 NSCLC

Consistent use of PET for non-invasive staging is a key component of work-up of patients with suspected/known NSCLC. However, minimally invasive confirmation is imperative given the imperfect accuracy of PET. Most studies reporting diagnostic performance of PET report findings on a per-patient basis; however, evidence presented above confirms the importance of station-by-station per nodal assessment in accurately defining extent of disease.

This practice is recommended by multiple Clinical Practice Guidelines/Expert Statements, including WABIP, BTS and AABIP [15,26].

#### 4.1.2. Consistent Sampling of All Observed LNs > 6 mm

As observed in the study by Craig and colleagues (above) the highest proportion of patients with false negative EBUS on staging of cN2a NSCLC had metastatic deposits > 3 mm [18], and reports on pathologic upstaging of cN0-resected NSCLC identify median size of metastatic deposit of under 8 mm. Consistent with this, the median size of LN identified with PET-occult metastases in the SEIMSIC study was 7 mm [19]. Therefore, there is not a size threshold below which sampling is obviated, and consistent with published guidelines, the practice of systematic EBUS mediastinal LN staging should involve examination of all mediastinal stations, with sampling of all LNs ≥ 6 mm.

#### 4.1.3. Reduce Variance in Care Through Use of Synoptic Procedural Reporting

Synoptic reporting is a process for reporting specific data elements in a specific format in surgical pathology reports, ensuring that all reports contain all necessary data elements, and facilitating performance monitoring as well as benchmarking at both individual proceduralist and institutional level [27]. Synoptic templates may be built directly from established clinical guidelines to ensure consistency of practice aligns with consensus regarding optimal care. In 2020, the Commission on Cancer (CoC) launched six new accreditation standards known as the CoC Operative Standards, with four of these standards (5.3–5.6) requiring cancer programmes across the US to include certain data elements in their operative reports in a synoptic format to aid performance monitoring and audit reporting [28].

A high variance in care is consistently noted regarding frequency of systematic endoscopic LN staging [13,14,29], despite this being a key quality indicator in guidelines describing quality indicators for performance monitoring in EBUS [15]. This includes both the proportion of patients undergoing systematic LN sampling and metrics such as the number of LNs sampled per procedure [16].

A novel pilot study reported in this Special Issue of *Cancers* examines the impact of synoptic procedural EBUS reports and notes significant improvements in quality of process measures (including mean number of LNs sampled and proportion of patients with at least one radiologically normal LN sampled), as well as increasing the rate of detection of PET-occult nodal metastases from 3.1% to 11.7% (*p* = 0.036) [12], which should be considered a key outcome measure. Synoptic reporting both encourages more consistent performance of systematic LN sampling during EBUS and enables reporting of these measures to inform performance monitoring, benchmarking and, where relevant, identification of underperformance to facilitate targeted interventions to improve performance and reduce variance in care.

#### 4.1.4. Regular Performance Monitoring, with Use of Quality Metrics Specific to Systematic Staging (For Example, e-R0)

Use of quality metrics has informed key improvements in both surgical and endoscopic management. It is essential that local performance is regularly measured against established benchmarks. Both the World Association of Bronchology and Interventional Pulmonology (WABIP) and the British Thoracic Society (BTS) have provided quality standards for staging EBUS that are aligned and consistent and provide a framework for quality assurance globally [15,30]. The BTS has recently completed a pilot audit of approximately 2000 EBUS procedures across nearly 30 centres [14] and demonstrated it was feasible to run a national audit of all UK hospitals which is progressing in 2026. It also provided insights into the critical importance of quality assurance programmes in EBUS. It demonstrated that 20% of staging EBUS procedures was not performed systemically and the sensitivity for nodal metastases fell below that minimum standards. This supports the implementation of the recommendations within this manuscript to optimize quality and performance.

While evidence remains limited, there are clear arguments for certain procedural/pathologic outcomes as measures of procedural quality specific to systematic endoscopic staging procedures. The rate of detection of PET-occult metastases is an objective outcome measure that is a self-evident quality marker given the immediate implications of such a finding; however, other process measures are perhaps more influential in achieving optimization of detection of PET-occult metastases. These include the number of LNs sampled per procedure, and the number of patients with at least one radiologically normal LN sampled. In the BTS pilot audit, 20% of individual lymph nodes sampled was judged to be inadequate during pathological assessment. Increasing the number of radiologically normal lymph nodes sampled should increase technical skill across all EBUS operators and help improve this important metric.

In patients with radiologically normal mediastinum, limited reports suggest the number of LNs sampled may improve accuracy of EBUS staging, although some variance in this finding may be related to the number of LN observed [21]. To ensure optimal procedural quality, including sampling of all LN ≥ 6 mm, we strongly encourage proceduralists to record the proportion of patients in whom radiologically normal LNs are sampled. This is designed in part to encourage as thorough sampling as possible. It will also permit assessment of the clinical impact of this practice and ascertainment of the validity of the concept of endoscopic R0 (as discussed above), which is yet to be established.

### 4.2. Preferred Where Resources Allow, Strongly Recommended for Consideration by EBUS Centres Where Performance Falls Below the Target Quality Standards as Quality Improvement Interventions

Variance in care may extend beyond individual procedural proficiency through impact of resource availability, or practice limitations due to jurisdiction-specific practice restrictions. We view the two proposed practice recommendations to be preferred but recognize not all centres will be able to institute these into routine EBUS practice. Where performance monitoring identifies sub-threshold outcomes in recognized quality markers (WABIP, BTS), institutions should strongly consider allocation of additional resources to facilitate these evidence-based practice steps as targeted interventions for quality improvement.

#### 4.2.1. Consider the Addition of EUS-B-FNA

Initial studies on minimally invasive ultrasound-guided staging of NSCLC were completed with an endoscopic ultrasound (EUS) scope via the esophagus and pre-date the first reports of EBUS staging [31]. Consequent studies confirmed the ability to perform EUS-guided fine needle aspiration (FNA) using the bronchoscopic videoscope (EUS-B) [32] and that EUS-B-FNA could be performed within the same procedure as EBUS-TBNA [33]. Meta-analysis confirms that addition of EUS-B to EBUS improves rate of detection of mediastinal LN metastases [16,34], with only very limited additional procedure duration. Interestingly, this is likely due to sampling of different regions of LN, as most additionally detected cases of metastases occur in LNs assessable by EBUs-TBNA—this is one key intervention to address the inherent false negative rate of EBUS, as described above in discussion of the paper by Craig and colleagues [18].

Systematic reviews of studies reporting outcomes of systematic staging in patients with cN0/1 NSCLC report a Number Needed to Treat (NNT) of 14 for EBUS for detection of one patient with N2 involvement, with NNT improved to seven by addition of EUS-B. Cusum analysis demonstrates that experienced EBUS proceduralists can safely and accurately perform EUS-B-FNA [35], with a high diagnostic sensitivity for lymph node assessment (and parenchymal lesions [36]). Where jurisdictional credentialing permits, this should be routinely incorporated into staging practice for more complete differentiation of cN2a from cN2b.

#### 4.2.2. Consider the Addition of ROSE to Staging EBUS Services

Rapid On-Site Evaluation (ROSE) is a method where the first part of an EBUS-TBNA specimen is immediately fixed to a slide and evaluated by a cytopathologist or pathology technician in the procedure room or in a remote location using digital pathology technology. Samples are assessed for adequacy and provide a preliminary diagnosis and immediate feedback to the bronchoscopist. ROSE has a number of potential benefits including improved staging sensitivity, improved diagnostic yield, shortened procedure time, lower sedation dose, fewer repeat procedures, and faster initiation of and higher rates of successful biomarker testing (e.g., genomic testing). However, in the BTS National Pilot Audit in EBUS in the UK, only 4% of procedures were supported by ROSE, which may reflect concerns over resource, cost and health economics. Recent data from the UK that compared 1650 EBUS procedures at a ROSE-supported EBUS service and a non-ROSE-supported EBUS service within the same geographical population reported statistically significant improvements in diagnostic performance with a 10% increase in sensitivity in staging EBUS and a 7% increase in sensitivity in diagnosis of advanced stage lung cancer [37]. ROSE may offer an opportunity to improve quality standards in EBUS and improve the differentiation of N2a and N2b, especially in services in which quality standards have not been met and resources and funding can be allocated to this intervention.

## 5. Summary

Routine use of minimally invasive staging via EBUS bronchoscopy has had a profound impact on the practice of lung cancer care. Systematic mediastinal staging is now considered standard of care in assessment of patients with early-stage disease. While less extensive evidence is published regarding systematic staging in patients with locally advanced disease, what is known consistently points towards the crucial role for accurate systematic LN evaluation to direct optimal planning, including accurate delineation of radiation fields, detection of occult N3 disease (which is likely to steer clinicians towards non-surgical modalities of care) and even down-staging of cN2 disease (based on PET) to cN0, therapy ensuring patients eligible for operative management are not erroneously denied curative intent resection.

The 9th edition TNM staging system delineation of N2 disease into cN2a and cN2b does not immediately have strong predictive value, though anecdotally may provide significant information that may guide treatment decision-making. Regardless, the 9th edition TNM system does emphasize the importance of accurate lymph node staging and is expected to drive refinements and improvements in both the consistency and quality of systematic endoscopic staging of NSCLC.

Meticulous and consistent performance of systematic endoscopic staging will be critical to individual patient assessment, and a reduction in variance in care and improvement in staging quality may be achieved through implementation of the proposal we present above. These proposals will require prospective studies to confirm their impact, which we keenly await.

## 6. Conclusions

Systematic endoscopic mediastinal lymph node staging is imperative to most accurately define the extent of LN involvement in patients with locally advanced (cN2) NSCLC. It provides the most accurate nodal information in comparison to imaging techniques and is the most likely investigation to differentiate N2a from N2b disease. While the differentiation between N2a and N2b is not strictly predictive, the prognostic value may support personalized treatment decision-making.

Accumulation of evidence to validate the prognostic and potentially the predictive value of accurately defining N2a and N2b N-status will be supported by more consistent and more comprehensive mediastinal endoscopic staging in this group, aided by more detailed (synoptic) reporting. We identify proposed solutions for enhancement of systematic staging in patients with N2 NSCLC, including routine performance of systematic lymph node assessment, with sampling of all LNs ≥ 6 mm, addition of EUS-B-FNA where possible, and use of synoptic reporting to reduce variance in care and enable performance monitoring.
